# Quercetin prevents osteoarthritis progression possibly via regulation of local and systemic inflammatory cascades

**DOI:** 10.1111/jcmm.17672

**Published:** 2023-02-01

**Authors:** Haiyan Wang, Yongyong Yan, Janak L. Pathak, Wei Hong, Jing Zeng, Dongyang Qian, Binwei Hao, Haiqing Li, Jinlan Gu, Richard T. Jaspers, Gang Wu, Ming Shao, Gongyong Peng, Haifeng Lan

**Affiliations:** ^1^ Affiliated Stomatology Hospital of Guangzhou Medical University, Guangdong Engineering Research Center of Oral Restoration and Reconstruction, Guangzhou Key Laboratory of Basic and Applied Research of Oral Regenerative Medicine Guangzhou China; ^2^ Laboratory for Myology, Department of Human Movement Sciences, Faculty of Behavioural and Movement Sciences Vrije Universiteit Amsterdam, Amsterdam Movement Sciences Amsterdam The Netherlands; ^3^ State Key Laboratory of Respiratory Diseases, National Clinical Research Center for Respiratory Diseases, National Center for Respiratory Medicine, Guangzhou Institute of Respiratory Health The First Affiliated Hospital of Guangzhou Medical University Guangzhou China; ^4^ GMU‐GIBH Joint School of Life Sciences Guangzhou Medical University Guangzhou China; ^5^ Liwan Central Hospital of Guangzhou Guangzhou China; ^6^ Department of Orthopaedics, The First Affiliated Hospital Guangzhou Medical University/Guangdong Key Laboratory of Orthopaedic Technology and Implant Materials Guangzhou China; ^7^ Department of Pulmonary and Critical Care Medicine, Shanxi Bethune Hospital Shanxi Academy of Medical Sciences Taiyuan China; ^8^ Department of Oral and Maxillofacial Surgery/Pathology, Amsterdam UMC and Academic Centre for Dentistry Amsterdam (ACTA) Vrije Universiteit Amsterdam, Amsterdam Movement Science Amsterdam The Netherlands; ^9^ Department of Orthopaedic Surgery The Third Affiliated Hospital of Guangzhou Medical University Guangzhou China

**Keywords:** inflammation, osteoarthritis, osteochondral degeneration, pro‐inflammatory markers, quercetin

## Abstract

Due to the lack of effective treatments, osteoarthritis (OA) remains a challenge for clinicians. Quercetin, a bioflavonoid, has shown potent anti‐inflammatory effects. However, its effect on preventing OA progression and the underlying mechanisms are still unclear. In this study, Sprague–Dawley male rats were divided into five groups: control group, OA group (monosodium iodoacetate intra‐articular injection), and three quercetin‐treated groups. Quercetin‐treated groups were treated with intragastric quercetin once a day for 28 days. Gross observation and histopathological analysis showed cartilage degradation and matrix loss in the OA group. High‐dose quercetin‐group joints showed failure in OA progression. High‐dose quercetin inhibited the OA‐induced expression of MMP‐3, MMP‐13, ADAMTS4, and ADAMTS5 and promoted the OA‐reduced expression of aggrecan and collagen II. Levels of most inflammatory cytokines and growth factors tested in synovial fluid and serum were upregulated in the OA group and these increases were reversed by high‐dose quercetin. Similarly, subchondral trabecular bone was degraded in the OA group and this effect was reversed in the high‐dose quercetin group. Our findings indicate that quercetin has a protective effect against OA development and progression possibly via maintaining the inflammatory cascade homeostasis. Therefore, quercetin could be a potential therapeutic agent to prevent OA progression in risk groups.

## 1 INTRODUCTION

Osteoarthritis (OA) is the most prevalent form of arthritis, which is the leading cause of disability among older adults and results in an inevitable economic and social burden on patients and families.^1^, ^2^ OA is generally characterized by progressive cartilage matrix destruction, subchondral bone sclerosis, and synovitis, resulting in pain, stiffness, and mobility loss.^3^, ^4^ Currently available therapeutic strategies for OA focus on relieving inflammation and pain.^5^ In addition to joint replacement surgery, OA is commonly considered an incurable disease. Therefore, exploring the therapeutic agents that mitigate OA progression is critical for OA treatment.

The pathophysiology of OA is complex. Several factors are known to contribute to joint damage in OA, such as proinflammatory cytokines (e.g., interleukin‐1 beta (IL‐1β), tumour necrosis factor‐alpha (TNF‐α) and IL‐6), matrix metalloproteinases (MMPs), and aggrecan (a disintegrin and metalloproteinase with thrombospondin motifs (ADAMTS)), which mediate extracellular matrix (ECM) degradation, as well as the inducible nitric oxide synthase‐nitric oxide (NO) system.^6^, ^7^, ^8^ Importantly, these factors interact closely with one another. For example, the production of MMP‐13 and ADAMTS‐5 can be efficiently induced by IL‐1β in OA, resulting in cartilage breakdown.^9^, ^10^ Levels of cytokines, chemokines, chemokine receptors, and growth factors in synovial fluid are elevated during OA development and progression. Most of these factors are also elevated in the serum of OA patients. Altered levels of cytokines, chemokines, chemokine receptors, and growth factors in synovial fluid and serum disrupt the inflammatory cascade homeostasis responsible for OA development and progression. Thus, additional approaches aimed at reducing the effects of inflammatory agents and ECM degradation may provide novel therapeutics for OA.

Several dietary polyphenols such as quercetin, resveratrol, curcumin, epigallocatechin‐3‐gallate, rosmarinic acid, genistein, ginger, berries, silver fir, pine bark, and others have shown pain‐relieving and anti‐inflammatory effects and in OA models.^11^ Quercetin, a flavonoid in many fruits, herbs, and vegetables, has shown anti‐inflammatory, immunomodulatory, and antioxidative effects.^12^, ^13^, ^14^, ^15^ Quercetin has antiarthritic properties and joint protective effects in various inflammatory joint diseases.^16^, ^17^ Recent studies indicated that quercetin alleviates OA pathogenicity by inhibiting inflammation and apoptosis of chondrocytes, promoting macrophage polarization to the M2 phenotype.^18^ The study also reported that quercetin inhibits endoplasmic reticulum stress‐related cartilage degeneration via activating SIRT1/AMPK signalling pathway and prevents the progression of OA in a rat model.^19^ Quercetin suppresses OA progression and reduces the levels of pro‐inflammatory cytokine IL‐1β, IL‐18, and TNF‐α in the OA rat model.^20^ However, the anti‐inflammatory activity of quercetin during OA development and progression is not fully understood. Few studies have investigated the modulatory effects of quercetin on local and systemic inflammatory cascades in OA.

The present study aimed to investigate the effect of quercetin treatment on OA progression and synovial and serum levels of inflammatory cytokines and growth factors in a rat OA model. Monosodium iodoacetate (MIA)‐injection in the rat joint was used as an OA inducer. Quercetin protected the MIA‐induced OA progression, cartilage and subchondral bone destruction, and synovial and systemic inflammation.

## 2 MATERIALS AND METHODS

### 2.1 Chemicals and reagents

Quercetin and MIA were purchased from Sigma‐Aldrich (St Louis, MO, USA). Quercetin was dissolved in normal saline. MIA was dissolved in water to make the stock solution and the working concentration was prepared by diluting the stock solution in PBS. A Bio‐plex rat cytokine 22‐plex assay kit (12005641) for TNF‐α, IL‐1α, IL‐1β, IL‐6, IL‐7, IL‐12p70, IL‐18, IL‐4, IL‐5, IL‐10, IL‐2, IL‐13, IL‐17, IFN‐γ, GRO/KC, MCP‐1, MIP‐1α, MIP‐3α, G‐CSF, M‐CSF, GM‐CSF, and VEGF was purchased from Bio‐Rad (Hercules, CA, USA). Antibodies against MMP‐3 (ab3523), MMP13 (ab39012), ADAMTS4 (ab180953) ADAMTS5 (ab180953), aggrecan (ab3523), collagen II (ab39012) were purchased from Abcam.

### 2.2 Animals

A total of 30 healthy Sprague–Dawley male rats 6 weeks old weighing 230–250 g were obtained from the Guangdong Medical Science Experiment Center. Visual observation of rats before the experiment showed no sign of illness or joint diseases. Rats were consuming food and water properly. Rats were housed under a controlled temperature (25 ± 2°C), a constant light cycle (12 h light/dark), and suitable humidity (45%–65%). All animals were allowed free access to food and water. The experimental procedure was started 7 days after adaptive feeding. All animal procedures were approved by the Committee of Animal Experiments of Guangzhou University of Chinese Medicine.

### 2.3 Induction of OA and experimental design

Rats were randomly divided into five groups (*n* = 6/group): control group, MIA (OA) group, and three quercetin‐treated groups (25, 50, and 100 mg/kg, i.g., q.d.). The healthy control group and OA group were used to confirm the OA development after MIA injection. Three doses of quercetin were used to find out the optimal concentration of quercetin to prevent OA development. Pieces of the literature showed the beneficial effect of 10–100 mg/kg oral or peritoneal administration of quercetin on OA treatment in different animal models.^19^, ^21^ Based on these reports from the literature, this study chooses 25, 50, and 100 mg/kg quercetin concentrations. The animals were anaesthetised by isoflurane (2.5%), and the depth of anaesthesia was checked by a toe pinch. Rats were treated with a single intra‐articular injection of 1 mg of MIA in the right knee.^22^ Rats in the control group were administered an equivalent volume of PBS. Rats in the quercetin‐treated groups received intragastric administration of quercetin from day 0 of MIA injection to day 27, once a day. The weight of the rats was measured every week. All rats were sacrificed on day 28, and the right knee cartilage tissues were collected. The joint surface morphology was macroscopically observed under the dissecting microscope.

### 2.4 Synovial fluid and serum collection

Rats were euthanized with sodium pentobarbital (100 mg/kg, i.p.) and 500 μl of PBS was injected into the rat knee articular joint cavity. The PBS was aspirated out after 5 times injections and withdrawal procedures. Then, the synovial fluid was centrifuged, and the supernatants were stored at −80 °C. Blood was collected from the abdominal aorta. After standing for 30 min at room temperature, the samples were centrifuged at 4500 RPM for 10 min and the serum was stored at −80°C.

### 2.5 Micro‐CT imaging

Micro‐CT was used to assess tibial subchondral bone surface and secondary osteoporosis. The knee joints were collected, and the attached soft tissue was removed thoroughly and fixed in 70% ethanol. Micro‐CT was performed with a micro‐CT (SkyScan 1172 Bruker micro‐CT NV, Kontich, Belgium) with an image pixel size of 10 μm, a source voltage of 60 kV, and a source current of 100 μA. Fifty slices of proximal tibia starting at 2 mm from the end of the growth plate were analysed. Bone mineral density (BMD), bone volume/total volume (BV/TV), bone surface density (BS/TV), trabecular number (Tb.N), trabecular separation (Tb.Sp), trabecular thickness (Tb.Th), trabecular pattern factor (Tb.Pf) were calculated separately as described previously.^23^

### 2.6 Histology

Following scanning, rat joints were washed and then transferred to 10% EDTA solution for 30 days for decalcification, and the solution was replaced every 3 days. Next, the decalcified tissues were dehydrated and embedded in paraffin, and 5‐μm thick sections were stained with haematoxylin and eosin (H&E), Masson, and safranin O/fast green. The histological evaluation was conducted using the OA Research Society International (OARSI) scoring system in a blinded manner.^24^

### 2.7 Immunohistochemistry

Briefly, after deparaffinization and rehydration through graded alcohol solutions, sections were used for antigen retrieval in 10 mM sodium citrate (pH 6.0) for 15 min and cooled to room temperature (RT). All the sections were washed with TBST, endogenous peroxidase was quenched with 3% hydrogen peroxide for 15 min, and the sections were washed with TBST. The sections were blocked with 5% donkey serum for an additional 2 h at RT before incubation with primary antibodies (MMP‐3, MMP‐13, ADAMTS4, ADAMTS5, aggrecan, and collagen II) overnight at 4°C followed by peroxidase‐labelled appropriate secondary antibodies for 30 min at RT. All sections were developed with diaminobenzidine (DAB) chromogen, followed by haematoxylin counterstaining, dehydration, and mounting. The semi‐quantification of the positive area was analysed using ImageJ software as described in the literature.^25^

### 2.8 Cytokines in the synovial fluid and serum of the joint

OA is characterized as an inflammatory disease, as various inflammatory cytokines are involved in the development and progression of OA.^26^ Cytokines were detected by Luminex‐based MILLIPLEX assay using a Bio‐plex Pro reagent kit with the Bio‐Plex suspension array technique according to the manufacturer's instructions. The levels of TNF‐α, IL‐1α, IL‐1β, IL‐6, IL‐7, IL‐12p70, IL‐18, IL‐4, IL‐5, IL‐10, IL‐2, IL‐13, IL‐17, IFN‐γ, GRO/KC, MCP‐1, MIP‐1α, MIP‐3α, G‐CSF, M‐CSF, GM‐CSF, and VEGF were analysed in the synovial fluid of the affected joint and serum by a Bio‐plex Pro rat cytokine 22‐plex assay kit (12005641). Serum and synovial fluid were centrifuged at 3000 rpm for 15 min, collected, and stored at −80°C until analysis. All samples were thawed on ice, diluted 4‐fold in serum, and diluted 2‐fold in synovial fluid of the joint for the final assay. The final concentrations of cytokines were calculated by Bio‐Plex Manager 6.1 software (Bio‐Rad) based on standard curves.

### 2.9 Statistical analysis

All data are presented as the mean ± standard deviation (SD). Data were analysed using one‐way analysis of variance (anova). Relative indices were analysed using SPSS version 22.0 software (SPSS). Data normal distribution was analysed by the Shapiro–Wilk test. LSD (homogeneous variances) and Dunnett's C (unequal variances) were used to perform post hoc tests for multiple comparisons. The data were graphically presented using GraphPad Prism 7 (GraphPad Software Inc.). Differences were considered statistically significant at a *p*‐value <0.05

## 3 RESULTS

### 3.1 Quercetin prevented OA progression

**FIGURE 1:**
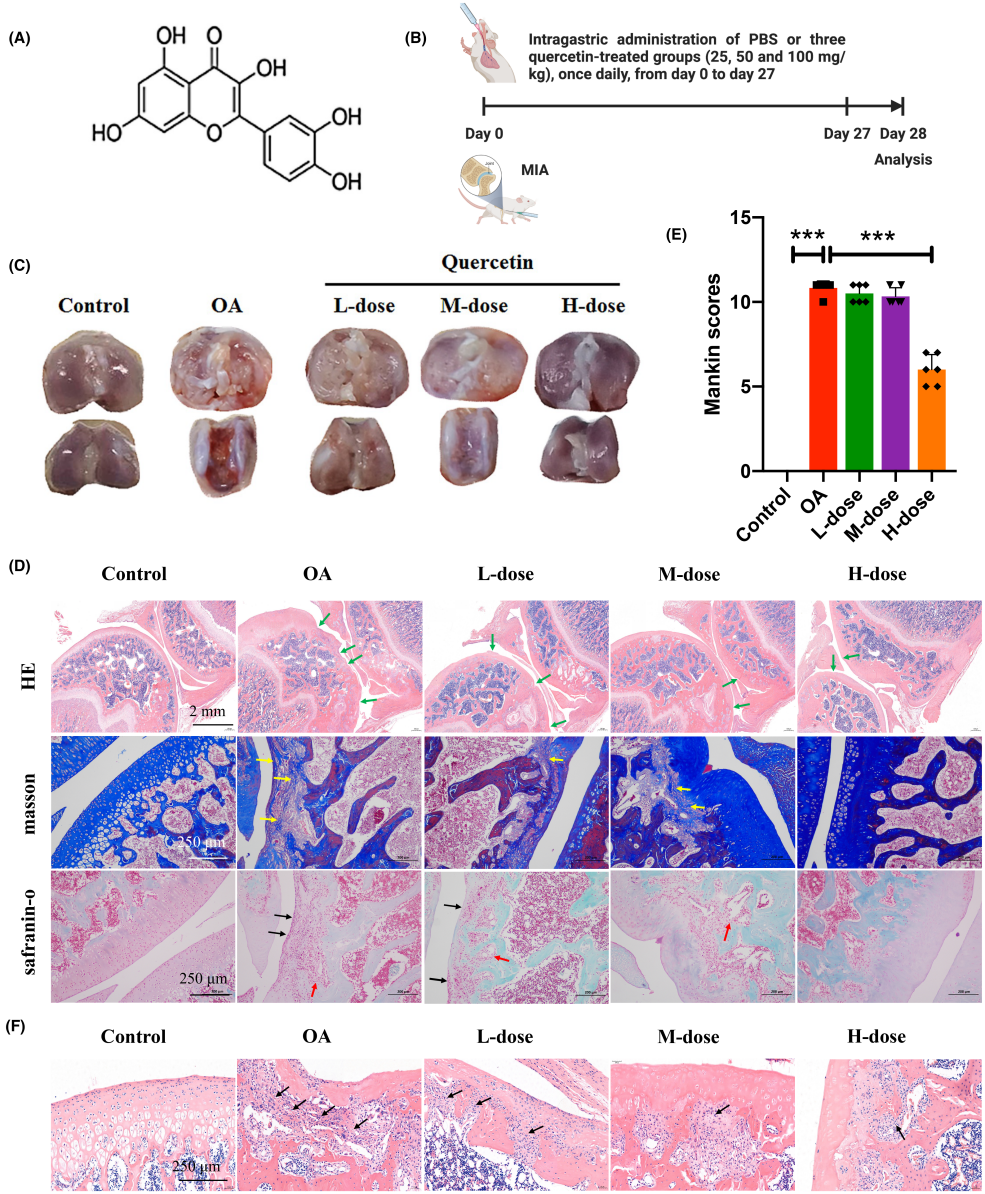
Quercetin mitigated OA progression. (A) Chemical structure of quercetin. (B) Timeline for the development and treatment process of MIA rats (the image was created with BioRender.com) (C) Macroscopic appearance of knee joints. (D) Representative H&E, Masson, and safranin O/fast green staining of cartilage in five groups. Green arrow, cartilage surface irregularities; yellow arrow, accumulation of collagen; black arrow, hyperplastic of cells in connective tissue; red arrow, myelofibrosis. (E) The degree of cartilage destruction was evaluated by Mankin scores. (F) Histological images showed infiltrated inflammatory cells (black arrow) in OA or/and quercetin‐treated rat joints. Data are presented as mean ± SD, *n* = 6. Significant differences between groups, ***p < 0.001.

The chemical structure of quercetin and the experimental design are listed in Figure 1A,B. No adverse effects, including body weight changes, were observed in MIA and/or quercetin injected into the animals throughout the experiments. Macroscopic evaluation of cartilage on knee joints showed that the articular cartilage in the control group presented with normal morphology and a smooth surface, while the OA group exhibited a clear reduction in the cartilage matrix, with an irregular morphological structure and rough surfaces. However, the low and medium‐dose quercetin group displayed a marked increase in articular cartilage thickness and improvements in the cartilage surface, and the cartilage damage was visibly alleviated (Figure 1C). In the high‐dose quercetin group, the new joint morphology and roughness were as good as in the control group.

The results from HE, Masson, and safranin O/fast green staining for each group are shown in Figure 1D. The control and high‐dose quercetin groups showed normal cartilage structures. In the OA group, the surface layer consisted of damaged cartilage, with reduced and irregular chondrocyte aggregation and inflammatory cell infiltration, and these degenerative changes extended to the deeper region. The low and medium‐dose quercetin groups displayed a marked better articular cartilage thickness and cartilage surface, with fewer numbers of inflammatory cell infiltrates compared to the OA group (Figure 1D–F)

Furthermore, to evaluate the degree of cartilage degradation, we used the Mankin score system, which scores structural damage, cellular abnormalities, and matrix staining.^27^ As presented in Figure 1E, the Mankin scores of the OA group were significantly higher than those of the control group, and the high‐dose quercetin group exhibited significantly lower Mankin scores than the OA group. These results indicate that quercetin could protect articular OA‐related cartilage damage.

### 3.2 Quercetin treatment reduced the levels of OA‐progression‐related cytokines, chemokines, and growth factors in synovial fluid and serum

**FIGURE 2:**
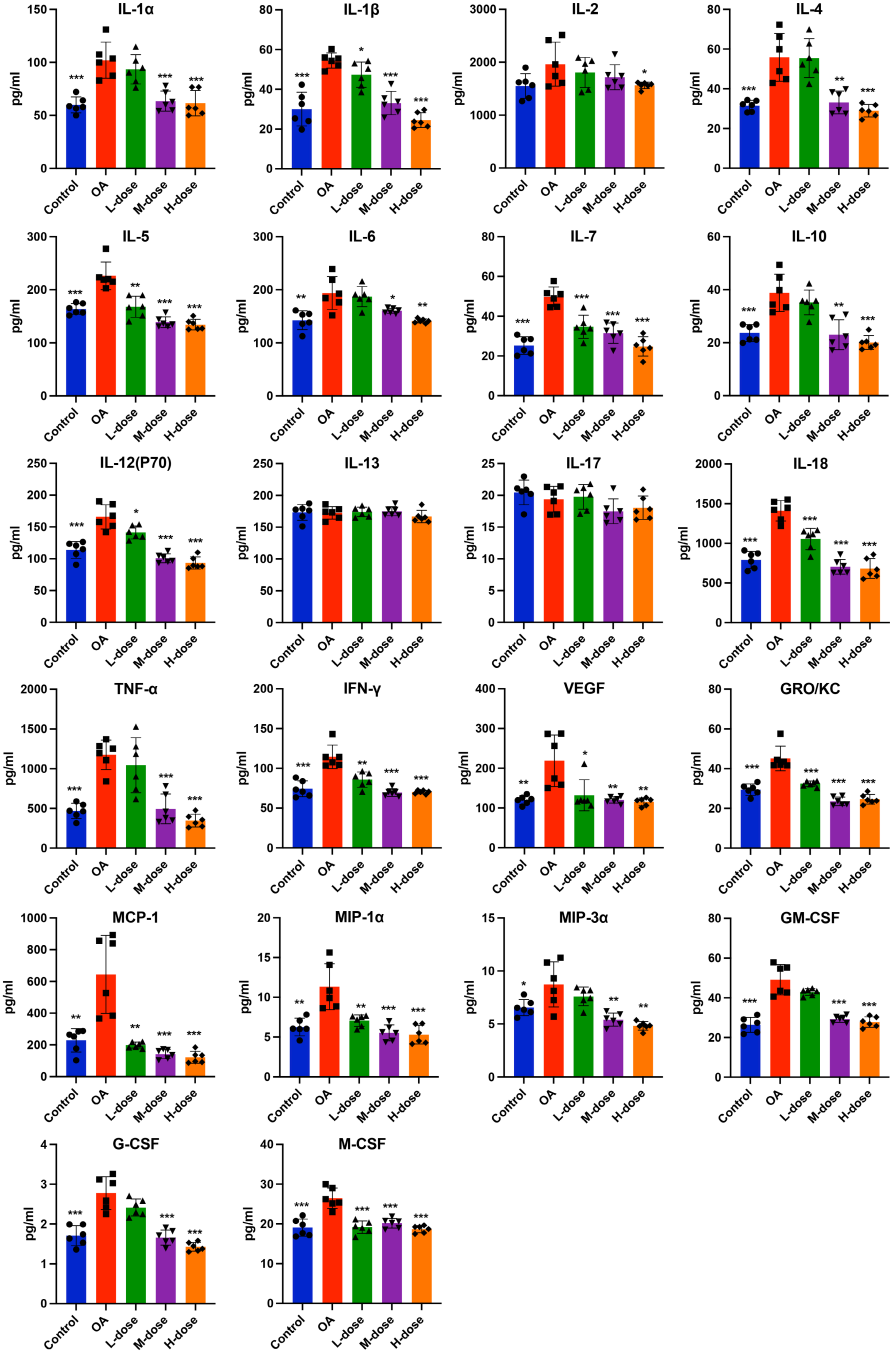
Quercetin inhibited levels of inflammatory mediators and growth factors in the synovial fluid of OA‐progressing joints. The expressions of pro‐ and anti‐inflammatory cytokines, chemokines and growth factors in the synovial fluid were analysed. The values are presented as the mean ± SD, *n* = 6. Significant difference compared to OA group, *p < 0.05, **p < 0.01, and ***p < 0.001.

**FIGURE 3:**
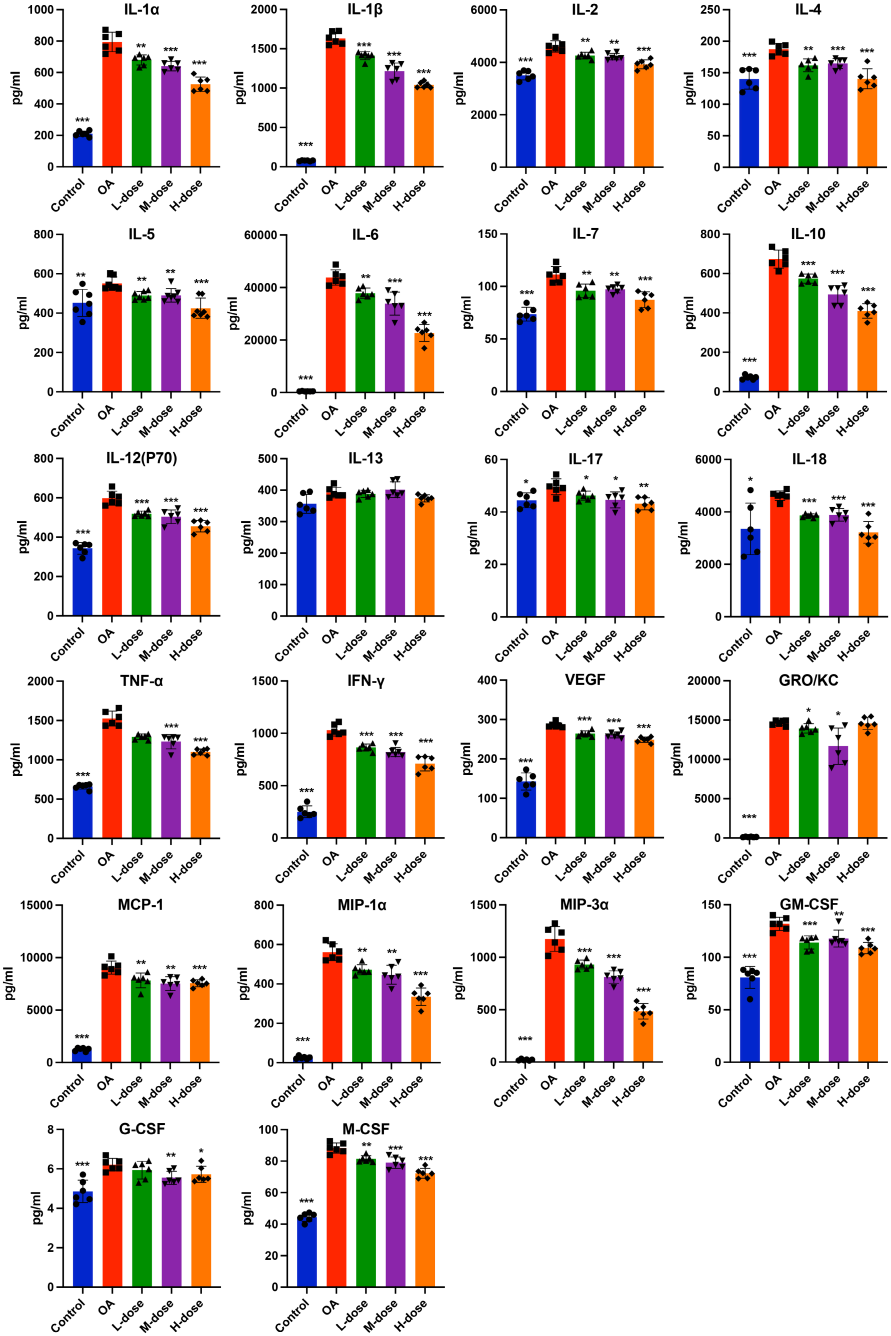
Quercetin inhibited levels of inflammatory mediators and growth factors in the serum of OA‐progressing rats. The expressions of pro‐ and anti‐inflammatory cytokines, chemokines and growth factors in the serum of each group were analysed. The values are presented as the mean ± SD, *n* = 6. Significant difference compared to OA group, *p < 0.05, **p < 0.01, and ***p < 0.001.

To investigate the anti‐inflammatory effects of quercetin on MIA‐treated rats, we evaluated cytokines in the serum and synovial fluid of joints when the rats were sacrificed. Elevated levels of inflammatory markers i.e., TNF‐α, IL‐1α, IL‐1β, IL‐6, IL‐7, IL‐12p70, IL‐18, IL‐4, IL‐5, IL‐10, IFN‐γ, GRO/KC, MCP‐1, MIP‐1α, MIP‐3α, G‐CSF, M‐CSF, GM‐CSF, and VEGF were found in the synovial fluid of joints of OA rats compared with those of control rats. However, these increases were reversed in the high‐dose quercetin group (Figure 2). The synovial levels of IL‐2, IL‐13, and IL‐17 remain unchanged in OA rats compared to the control (Figure 2). The serum levels of the majority of the cytokines, chemokines, and growth factors tested except IL‐13 were elevated in OA rats compared to control (Figure 3). These increases were reduced in the high‐dose quercetin group except for the level of GRO/KC. These data demonstrate that quercetin is capable to reduce the complex inflammatory cytokine environment in OA‐progressing rats. Medium‐dose quercetin treatment also reduced the majority of OA‐induced cytokines, chemokines, and growth factors in synovial fluid. But, low‐dose quercetin treatment only reduced the few OA‐induced cytokines, chemokines, and growth factors in synovial fluid (Figure 2). A similar effect of low and medium‐dose quercetin was observed in the serum‐level expression of cytokines, chemokines, and growth factors (Figure 3).

### 3.3 Quercetin downregulated the levels of MMP‐3, MMP‐13, ADAMTS4, and ADAMTS5 in OA‐progressing joints

**FIGURE 4:**
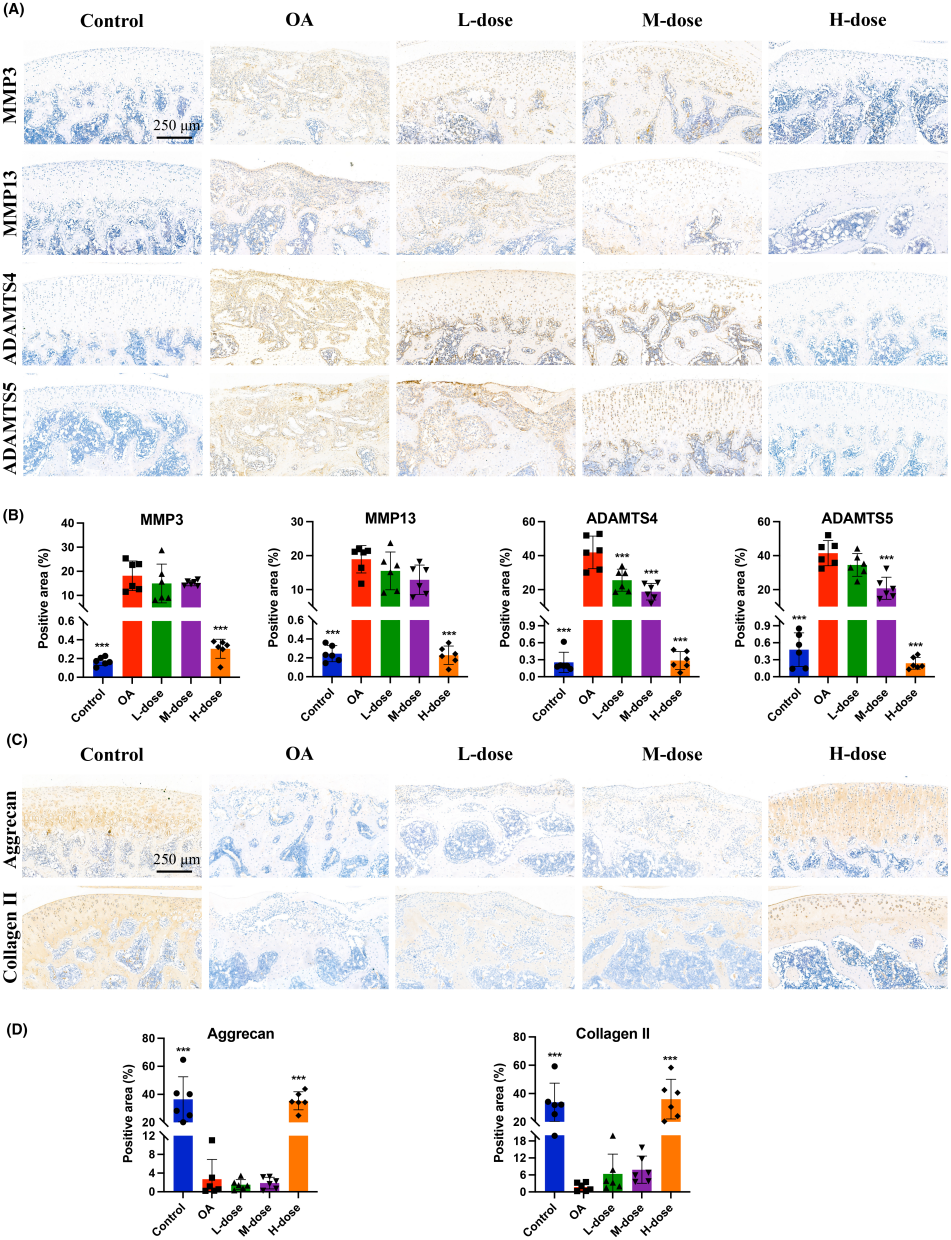
Quercetin downregulated the levels of MMP‐3, MMP‐13, ADAMTS4, and ADAMTS5, and retained the ECM contents in OA‐progressing joints. (A) Immunohistochemical staining was performed for the expression of MMP‐3, MMP‐13, ADAMTS4, and ADAMTS5 in the articular cartilage (the pictures shown are representative staining in each group). The brown colour showed positively stained cells for MMP‐3, MMP‐13, ADAMTS4, and ADAMTS5, respectively. (B) The semi‐quantification of the positive area of MMP‐3, MMP‐13, ADAMTS4, and ADAMTS5 expressions in rat cartilage. (C) The articular cartilage was immunostained for the expression of aggrecan and collagen II. The brown colour showed positively stained cells for aggrecan and collagen II, respectively. (D) The semi‐quantification of the positive area of aggrecan and collagen II expressions in rat cartilage. The values are presented as the mean ± SD, *n* = 6. Significant difference compared to OA group, *p < 0.05, **p < 0.01, and ***p < 0.001.

Proteins involved in the degradation of the extracellular matrix (ECM), including MMP‐3, MMP‐13, ADAMTS4, and ADAMTS5, which are important cartilage catabolic factors, were analysed by immunohistochemistry. We observed obvious upregulation of the protein levels of MMP‐3, MMP‐13, ADAMTS4, and ADAMTS5 in the OA model group compared to the control group. In contrast, high‐quercetin treatment suppressed MMP‐3, MMP‐13, ADAMTS4, and ADAMTS5 protein expression (Figure 4A,B).

### 3.4 Quercetin retained the expression of aggrecan and collagen II in OA‐progressing joints

Decreases in aggrecan and collagen II result in the compression or calcification of the ECM, followed by the acceleration of arthrosis.^28^ To assess the effects of quercetin on MIA‐treated rats, the protein levels of aggrecan and collagen II were evaluated. As shown in Figure 4C,D, compared with the control rats, OA rats exhibited significant downregulation of aggrecan and collagen II protein expression. However, the high‐dose quercetin treatment retained the levels of aggrecan and collagen II in OA‐progressing joints.

### 3.5 Quercetin prevented subchondral bone damage and bone loss in OA‐progressing joints

**FIGURE 5:**
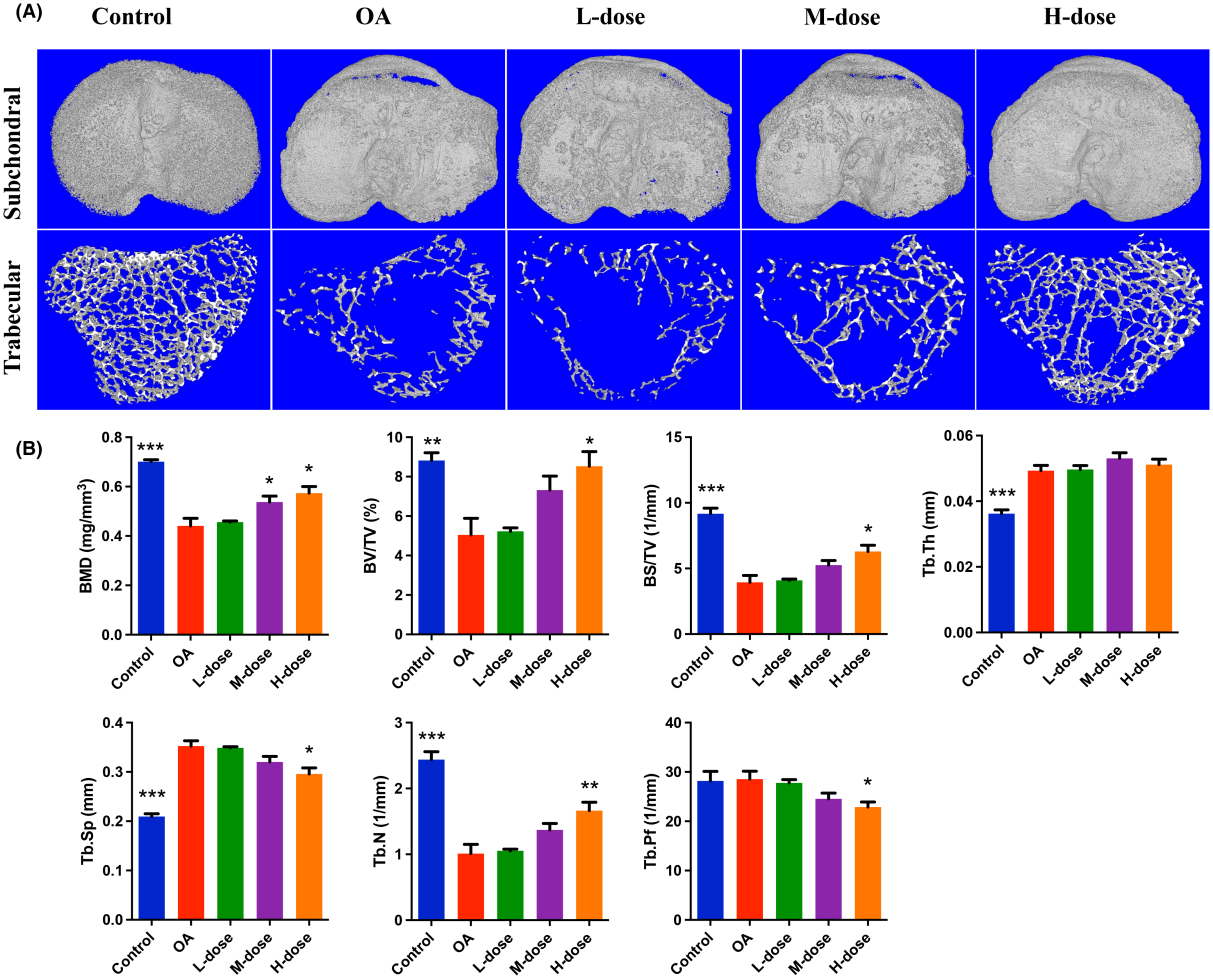
Quercetin prevented subchondral bone damage and bone loss in OA‐progressing joints. (A) Micro‐CT images of subchondral bone. (B) Quantification of trabecular bone parameters from micro‐CT images. The values are presented as the mean ± SD, *n* = 6. Significant difference compared to OA group, *p < 0.05, **p < 0.01, and ***p < 0.001.

The OA group showed significant subchondral bone injury accompanied by massive osteophyte formation compared with the control group (Figure 5A). High‐dose quercetin treatment effectively alleviated the destruction of subchondral bone and reduced the formation of osteophytes. Similarly, the trabecular bone of the OA group was significantly decreased compared with the control group which was reflected by the decreased BMD, BV/TV, BS/TV, Tb.N, and increased Tb.Th and Tb.Sp (Figure 5B). As the results showed, BMD, BV/TV, BS/TV, Tb.N increased and Tb.Sp decreased in the quercetin treatment group. Moreover, the Tb.Pf value was lower in the quercetin treatment group than in the OA group. These results indicated that quercetin treatment can effectively protect subchondral bone damage in OA‐progressing joints.

## 4 DISCUSSION

**FIGURE 6:**
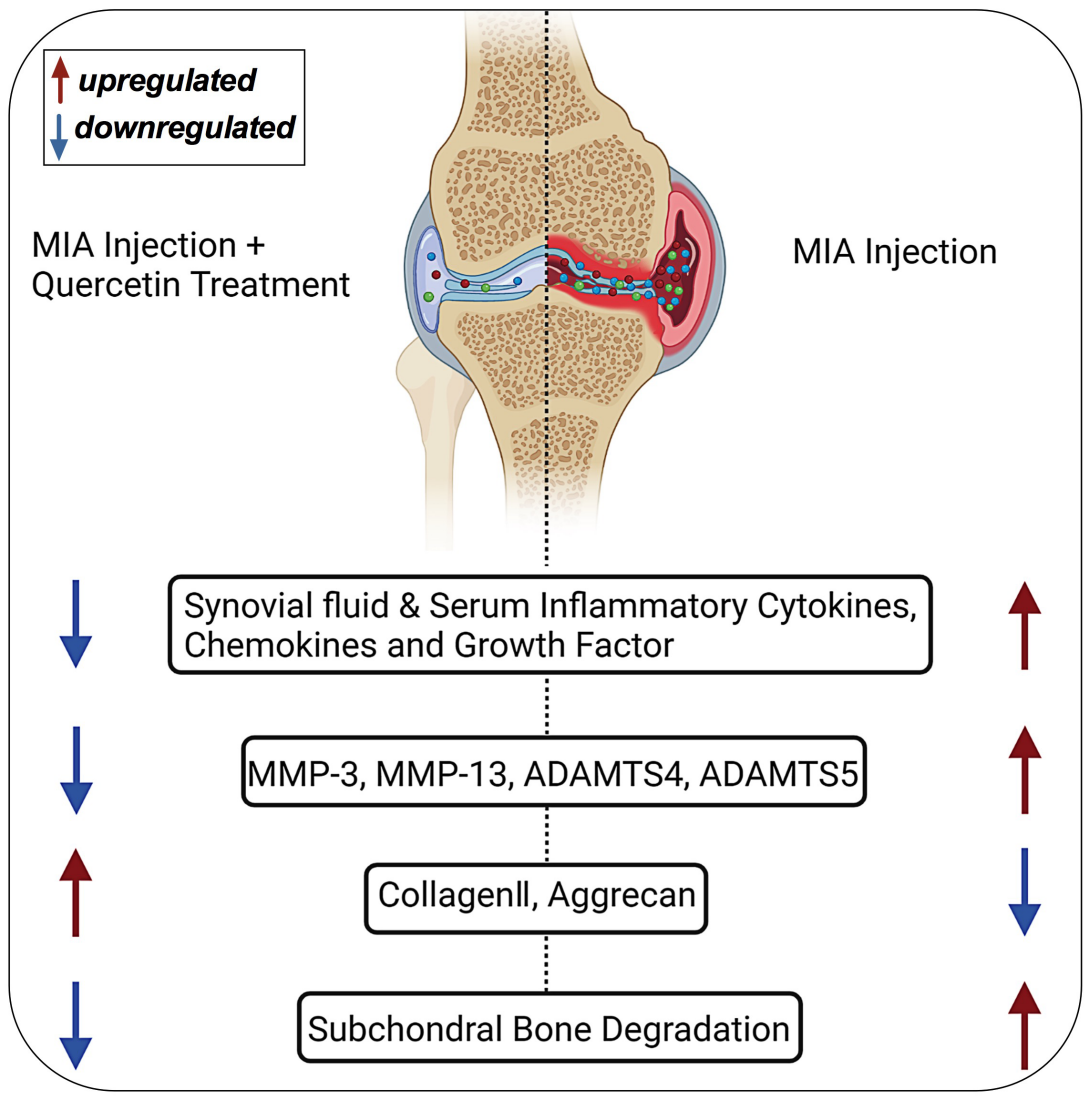
Scheme showing the protective effect of quercetin on OA progression in MIA‐induced rats. MIA induced the pathophysiology of arthritis including the expression of cytokines and growth factors, and quercetin treatment alleviated the effects of MIA. The image was created with BioRender.com

OA is the most prevalent arthritic disease that affects the joints. OA is an age‐related degenerative disease with a complicated pathology involving inflammation and ECM degradation. The majority of available therapies focus on relieving symptoms, but there are challenges to slowing the progression of the disease. Therefore, it is important to find new therapeutic agents to mitigate the development and progression of OA in vulnerable patients.^29^ Quercetin has been demonstrated to exert anti‐inflammatory activity in multiple disease models.^30^, ^31^, ^32^ However, the anti‐inflammatory activity of quercetin during OA development and progression is not fully understood. The administration of quercetin alleviated inflammation and ECM degradation thereby preventing OA progression in MIA‐treated rats (Figure 6). Our results indicate that quercetin prevents OA progression in MIA‐treated rats via mitigation of local and systemic inflammatory cascades.

An MIA intra‐articular injection is a widely used method to mimic the structural and functional changes that occur in human OA.^22^ Intra‐articular injection of MIA disrupts cellular glycolysis and induces cell death, which triggers local acute inflammation and subsequent cartilage degeneration. Synovitis, cartilage loss in knee joints, and subchondral bone damage are key features of OA.^33^ MIA injected rats showed cartilage loss, and a higher Mankin score indicating knee damage in OA rats. The quercetin‐treated rats showed intact cartilage, and a lower Mankin score compared to MIA alone‐treated rats. This indicates the protective effect of quercetin on MIA‐induced OA progression.

It is clear that inflammation is not only present in the majority of OA patients but also actively involved in the progression of the disease.^34^ Histological images showed a higher number of infiltrated inflammatory cells in OA joints and a high dose of quercetin treatment reduced the number of infiltrated inflammatory cells in joints. Infiltrated inflammatory cells are the key player in OA pathophysiology.^35^ Therefore, the inhibition of inflammatory cell infiltration in joints might be associated with quercetin‐mediated protection against OA development. The expression of proinflammatory cytokines and chemokines is a major event in the pathogenesis of OA.^36^ Reducing the increased expression of inflammatory mediators is considered a promising avenue for the treatment and prevention of OA. During the progression of OA, proinflammatory cytokines such as IL‐1β, IL‐1α, IL‐6, VEGF, IFN‐γ, and TNF‐α play critical roles in the pathogenesis of OA, which contribute to inflammation‐associated cartilage degradation.^37^, ^38^ IL‐7, IL‐10, IL‐12, IL‐13, and IL‐18 are upregulated in synovial fluid of OA patients.^39^ Elevated serum levels of IL‐2, IL‐4, IL‐5, IL‐17, G‐CSF, GM‐CSF, and other proinflammatory cytokines had been observed in the guinea pig OA model,^40^ suggesting the role of these cytokines and growth factors in OA. We found the simultaneous upregulation of pro‐ and anti‐inflammatory cytokines in OA synovial fluid and serum. Bastiaansen‐Jenniskens et al.^41^ summarized the simultaneous upregulation of pro‐, and anti‐inflammatory cytokines in OA synovial fluid and serum reported in various studies. The upregulation of anti‐inflammatory cytokines in OA synovial fluid and serum might be the effect of the body's compensatory mechanism to counteract the pro‐inflammatory environment. Mouse homologues of human growth‐related oncogenes/keratinocyte chemoattractant (GRO/KC) produced by chondrocytes, contribute to the pathophysiology of OA.^42^ Synovial levels of all the above‐mentioned cytokines, chemokines, chemokine receptors, and growth factors are elevated in OA and had been reported to play role in rat metabolic OA.^43^ Chemokines and chemokines receptors including MCP‐1, MIP‐3α, and MIP‐3α are upregulated in the joint and serum of patients with OA.^44^, ^45^ The immune cell trafficking role of chemokines and chemokines receptors has demonstrated a diverse range of effects on OA development and progression.^44^ MIA‐induced OA rats' synovial fluid showed elevated levels of all aforementioned cytokines, chemokines, chemokine receptors, and growth factors except IL‐2, IL‐13, and IL‐17. Similarly, all these factors except IL‐13 were elevated in OA serum. Quercetin treatment prevented the upregulation of factors in MIA‐treated rats. Our results indicate that quercetin has the potential to maintain inflammatory cascade homeostasis that prevents the development and progression of OA.

Current therapeutic agents alter the OA pathogenesis by targeting the regulation of inflammatory processes or the expression of growth factors or aggrecans.^46^, ^47^ Articular cartilage, an avascular tissue that forms the articulating surface of all joints, possesses an ECM to support normal biomechanics and homeostatic properties of joint cartilage. Cartilage is unique due to its limited regenerative ability and avascular nature. Thus, upon damage or destruction of joint cartilage, it is difficult to self‐repair. MMPs and ADAMTSs belong to the metzincins superfamily, characterized as zinc‐dependent enzymes responsible for ECM protein turnover, type II collagen degradation, and aggrecan activity.^48^ Altered levels of MMPs and ADAMTSs have been described in OA patients. Among the MMPs involved in OA, MMP‐13, mainly released by chondrocytes, is essential in successive phases of ECM remodelling.^7^ The role of MMP‐13 is crucial for OA development and progression, as it is the main factor involved in the degradation of type II collagen, along with other ECM components, including aggrecan. In addition to articular cartilage degradation, MMP‐13 is also involved in synovial inflammation, inducing synovial hyperplasia with mononuclear cell infiltration in the joint.^49^, ^50^ Clinical investigation revealed that patients with articular cartilage destruction have high MMP13 expression.^51^ MMP13 is hardly detected in normal adult tissues, suggesting that increased MMP13 may be associated with cartilage degradation. MMP‐13 is also present in synovial fluid from OA patients, where cytokines such as IL‐1β and other inflammatory mediators are involved in the induction of its expression.^52^ MMP‐3 is one of the MMPs most highly expressed in OA cartilage. The ADAMTS family of aggrecanases also contributes to proteoglycan/aggrecan depletion and is associated with cartilage degradation during OA. ADAMTS4 and ADAMTS5 are identified as the major aggrecanases during OA development. However, there is still a debate about which aggrecanase plays a major role in OA mainly due to discrepancies in results from murine OA models and human OA.^53^, ^54^ In murine OA models, ADAMTS5 is the major aggrecanase.^55^ In addition, ADAMTS4 is overexpressed in human OA but ADAMTS5 expression remains similar in synovial fibroblasts of both healthy donors and OA patients.^54^ Proinflammatory mediators, such as IL‐1β or TNFα, induce the expression of ADAMTS4 and ADAMTS5.^56^ Our results revealed significant upregulation of MMP‐13, MMP‐3, ADAMTS4, and ADAMTS5 in OA joints and quercetin treatment decreased their expressions indicating the role of quercetin in dampening the expression of MMPs and ADAMTSs in OA‐progressing joints.

Aggrecan is a high‐molecular‐weight proteoglycan that has a pivotal role in cartilage structure and the joints' functions. Aggrecan is one of the main components of the cartilage ECM, which also protects collagen from degradation. The loss of ECM aggrecans in the early stages of OA is one of the critical events in the development of the disease.^57^ The major structure of the ECM is composed of aggrecan and collagen type II, which preserve the normal physiological functions of cartilage. Loss of aggrecan and collagen type II contributes to the acceleration of OA progression. Thus, inhibition of the degradation of aggrecan and collagen type II may represent a novel treatment for OA.^58^ Our results indicate a novel role of quercetin in preserving cartilage integrity by rescuing type II collagen and aggrecan from MIA‐induced degradation.

Quercetin, a single extract natural product, has anti‐inflammatory properties and has shown potential to treat various inflammatory diseases.^59^ Quercetin is a senolytic drug that induces apoptosis of senescent cells.^60^ Senescent cells are associated with inflammation and tissue destruction. Therefore, the anti‐inflammatory and mitigation of OA‐induced tissue degeneration effects of quercetin could be associated with its senolytic property. Previous studies had reported the role of quercetin in the treatment of fully developed OA.^18^, ^19^, ^21^, ^61^ In this study, we investigated the effect of quercetin on the prevention of OA progression and its role in the homeostasis of OA pathogenicity‐related inflammatory cascades. This is the first study to evaluate the synovial and serum levels of 22 cytokines, chemokines, chemokine receptors, and growth factors in OA rats and quercetin‐treated OA‐progressing rats. Three doses of quercetin were used to evaluate the dose‐dependent protective effect against OA development. The limitation of this study is the lack of a mechanism clarifying how quercetin prevents OA progression. However, the OA joint is a complex microenvironment with the involvement of different cell types interacting with each other. The majority of previous studies have investigated the therapeutic effect of quercetin in OA via the effect on chondrogenic cells.^18^, ^19^ Sirse et al. used the primary human mesenchymal stromal cells (MSCs) from OA patients and healthy donors to determine the effects on the chondrogenesis of quercetin in vitro under normal and inflammatory conditions.^62^ Britti et al. showed that quercetin mixed with palmitoylethanolamide decreases inflammation and relieves pain in two different rat models of inflammation and OA.^63^ Our results also indicated that chondro/osteoprotective, anti‐inflammatory, and immunomodulatory effects of quercetin are mainly responsible for mitigating OA progression. Our research group is currently developing an in vitro model of osteoarthritis called joint in a chip, which is an in vitro chip platform that contains the majority of cells present in the joint including chondrogenic cells, synovial cells, macrophages, BMSCs, etc. Once this model is fully developed and optimized, it is possible to investigate the mechanism of quercetin‐based effect on different cells present in the joints during OA progression. The incidence of OA is increasing globally with the increase in the old age population. The current advancement in genetics can partly predict the risk group population that can develop OA. Quercetin has senolytic, anti‐inflammatory, and anti‐aging properties. Interestingly, our study showed that quercetin prevents OA development and progression. Therefore, quercetin could be a therapeutic agent to prevent OA development and progression in the risk group population.

## 5 CONCLUSION

Quercetin showed anti‐degenerative and anti‐inflammatory effects in OA‐progressing joints. Quercetin prevented cartilage damage and subchondral bone degeneration in OA progressing joints. MMPs and ADAMTSs levels were reduced and Collagen II and aggrecan levels in joint cartilage were maintained in quercetin‐treated OA progressing rats. Moreover, quercetin prevents the elevation of the majority of OA pathogenesis‐related cytokines, chemokines, chemokine receptors, and growth factors in synovium and serum of OA‐progressing rats. Our results indicate that quercetin as a potent therapeutic agent to prevent the development and progression of OA in risk groups.

## AUTHOR CONTRIBUTIONS

**Haifeng Lan:** Conceptualization (equal); funding acquisition (equal); project administration (lead). **Haiyan Wang:** Data curation (equal); investigation (equal); writing – original draft (equal). **Yongyong Yan:** Data curation (equal); investigation (equal); writing – original draft (equal). **Janak L. Pathak:** Methodology (equal); writing – review and editing (lead). **Wei Hong:** Formal analysis (equal); funding acquisition (equal). **Jing Zeng:** Resources (equal). **Dongyang Qian:** Funding acquisition (equal). **Binwei Hao:** Formal analysis (equal). **Haiqing Li:** Formal analysis (equal). **Jinlan Gu:** Investigation (equal). **Richard T. Jaspers:** Writing – review and editing (supporting). **Gang Wu:** Writing – review and editing (supporting). **Ming Shao:** Methodology (equal); supervision (equal). **Gongyong Peng:** Methodology (equal); supervision (equal).

## FUNDING INFORMATION

This work was supported by Ph.D. Initiation Project of the Third Affiliated Hospital of Guangzhou Medical University (No. 2018B14), Guangdong Medical Research Foundation (No. A2022253), Characteristic Innovation Projects of Universities in Guangdong Province (No. 2019KTSCX139), Open Project of the State Key Laboratory of Respiratory Disease (No. SKLRD‐Z‐202103), Science and technology innovation project of Guangzhou Medical University (No. 202102080045), Natural Science Foundation of Guangdong (No. 2021A1515012424) and Youth Innovative Talents Projects of Universities in Guangdong Province (No. 2019KQNCX121).

## CONFLICT OF INTEREST

The authors declare no conflict of interest.

## Data Availability

The data that support the findings of this study are available from the corresponding author upon reasonable request.
